# Beyond cardiac fibroblasts: research advances on understanding and targeting intercellular communication networks in cardiac fibrosis

**DOI:** 10.3389/fcvm.2026.1858990

**Published:** 2026-07-01

**Authors:** Xiduo Zhao, Lianyu Hua, Youjun Xiong, Fuyou Shi, Yilin Zhong, Shangwei Zhang, Chengnan Tian

**Affiliations:** 1 The First Clinical College, Gannan Medical University, Ganzhou, Jiangxi, China; 2 Department of Cardiovascular Surgery, The First Affiliated Hospital of Gannan Medical University, Ganzhou, Jiangxi, China

**Keywords:** extracellular vesicles, immune cells, intercellular communication, myocardial fibrosis, paracrine signaling, therapeutic targets

## Abstract

Cardiac fibrosis is pivotal in the progression of various cardiovascular diseases, ultimately leading to heart failure. Traditionally, research has focused on the activation and transformation of cardiac fibroblasts. However, the heart's complexity, involving multiple cell types, means fibrosis is not driven by a single cell type but by a network of intercellular communication. This network includes signaling mediators from the pericardial space, a crucial regulatory compartment for intercellular cross-talk. This article reviews recent advances that surpass the traditional single-cell approach, providing an in-depth analysis of the complex networks involved in myocardial fibrosis. These networks encompass cardiomyocytes, endothelial cells, and immune cells like macrophages and T cells, which interact through paracrine signaling, direct cell-cell contact, and extracellular vesicle-mediated mechanisms. The review delves into the molecular mechanisms of these communication modes, clarifying the functional outcomes of each signaling pathway in fibrosis initiation and persistence. It also summarizes the phenotypic changes and functional impairments caused by abnormal intercellular crosstalk, such as excessive extracellular matrix deposition, myocardial stiffness, electrical signal disturbances, and cardiac contractile dysfunction. Additionally, the review examines the latest therapeutic strategies and potential intervention targets, focusing on specific cytokines, signaling pathways, or vesicular delivery systems. It discusses the translational value of preclinical findings, the limitations of single-target interventions, and the need for multi-target combined strategies. The article concludes with insights into core regulatory nodes and future optimization directions for anti-fibrotic therapy. This exploration aims to provide new theoretical foundations and directions for developing precise and effective anti-fibrotic therapies.

## 1 Introduction

Cardiac fibrosis plays a crucial role in the progression of various heart diseases leading to heart failure. It is primarily characterized by the excessive deposition and remodeling of extracellular matrix components, such as collagen, within the cardiac stroma (1). Traditionally, myofibroblasts, which are activated cardiac fibroblasts, were thought to be the main contributors to matrix synthesis and secretion (2). However, advancements in technologies like single-cell sequencing have shifted research from focusing solely on individual effector cells to examining the complex intercellular communication networks within the heart. Recent evidence suggests that fibrosis is not driven solely by fibroblasts. Instead, it results from the coordinated regulation by cardiomyocytes, endothelial cells, and immune cells, including macrophages and lymphocytes, through intricate signaling networks (3). These networks convey pro-fibrotic or anti-fibrotic signals through various mechanisms, such as soluble factors, extracellular vesicles, gap junctions, and cell-extracellular matrix interactions. This complex signaling underpins the pathological development of fibrosis (4).

Understanding the complex communication network is crucial for identifying specific intervention targets in fibrosis. In diabetic cardiomyopathy, single-cell RNA sequencing reveals that fibroblasts, along with macrophages, endothelial cells, and epicardial cells, contribute to a pro-fibrotic microenvironment. This is facilitated by ligand-receptor interactions such as PDGF(s)-PDGFRα and EFEMP1-EGFR (5), leading to sustained fibroblast activation, increased collagen secretion, and microvascular dysfunction. These processes ultimately cause ventricular wall thickening and diastolic dysfunction. Similarly, in stress-induced hypertrophic cardiomyopathy, cardiac fibroblasts secrete proteins like PTN. These proteins interact with the SDC4 receptor, promoting fibroblast proliferation and invasion and triggering macrophage inflammatory responses, which collectively exacerbate myocardial fibrosis (6). The result of this crosstalk is progressive myocardial hypertrophy, expanded interstitial fibrosis, and reduced cardiac compliance, severely impairing cardiac pump function and increasing heart failure risk. These insights shift the perspective from viewing fibroblasts as isolated entities to recognizing the importance of intercellular communication.

The extracellular matrix (ECM) is not only the endpoint of fibrosis but also a crucial medium for intercellular communication and signal transduction (7). It provides a structural and biochemical framework for cells, storing signaling proteins such as growth factors that influence cardiac muscle cells, including fibroblasts (3). Under pathological conditions, excessive ECM deposition and abnormal remodeling alter its mechanical and biochemical properties. These changes impact cellular signaling through integrin mechanosensors, creating pro-fibrotic positive feedback loops (8). For example, the interaction between the TGF-β1 signaling pathway and the mechanosensitive transcription factor YAP is a key mechanism promoting cardiac myofibroblast activation and matrix stiffening (8). This synergy results in sustained myocardial stiffening, impaired cardiomyocyte contraction and relaxation, disrupted electrical conduction, and arrhythmogenesis, leading to irreversible pathological remodeling. Thus, dynamic changes in the ECM are crucial in regulating cellular communication networks.

Immune cells, particularly macrophages, play a vital role in the fibrotic signaling network (9). Omics-integrated analyses reveal that in myocardial fibrosis, which results from dilated cardiomyopathy and myocardial infarction, macrophages contribute to fibrosis through abnormal RNA metabolism and transcriptional dysregulation (10). Beyond cellular sources, the pericardial space acts as a unique signaling microenvironment, abundant in cytokines, chemokines, growth factors, and extracellular vesicles (11). Upon cardiac injury, mediators in the pericardial fluid are swiftly released, diffusing into the myocardial interstitium (12). They directly influence the interactions between cardiomyocytes, fibroblasts, endothelial cells, and immune cells, thereby initiating and amplifying fibrotic responses (3). This phenotypic shift and transdifferentiation lead to significant myofibroblast accumulation, excessive extracellular matrix (ECM) buildup, and scar formation (13). These changes result in ventricular dilation and decreased ejection fraction in dilated cardiomyopathy, as well as hindered cardiac repair following myocardial infarction. Consequently, atrial interstitial fibrosis, a shortened effective refractory period, and disorganized electrical activity emerge, forming the core mechanisms behind the initiation and maintenance of atrial fibrillation. These findings illustrate that immune cells extensively interact with fibroblasts and cardiomyocytes by secreting cytokines and growth factors, collectively affecting fibrosis outcomes.

In summary, myocardial fibrosis is a pathological process characterized by the interaction of various cardiac cell types through a complex communication network. Understanding the roles of these cells, along with the signaling patterns between them—such as ligand-receptor interactions, vesicular transport, and mechanical signaling—and their spatiotemporal dynamics, is essential for addressing therapeutic challenges and achieving precise interventions. This article explores this emerging paradigm by systematically discussing the latest research advances on the core components, regulatory mechanisms, and targeted intervention strategies of intercellular communication networks in myocardial fibrosis.

## 2 Literature retrieval methodology and selection criteria

This review utilized a narrative framework combined with systematic retrieval strategies to ensure comprehensive and timely inclusion of literature. The search was conducted in PubMed, Web of Science, Embase, and Scopus databases, encompassing publications from January 2018 to January 2026. Search terms included combinations of MeSH terms and keywords: “Myocardial fibrosis,” “Intercellular communication,” “Paracrine signaling,” “Extracellular vesicles,” “Immune cells,” and “Therapeutic targets.” The inclusion criteria were: (1) Original research articles (*in vivo*, *in vitro*, omics studies) and high-quality reviews focusing on intercellular communication networks in cardiac fibrosis; (2) Studies published in peer-reviewed journals with complete experimental design and clear conclusions; (3) Literature written in English for consistency and accessibility. Exclusion criteria included: (1) Studies unrelated to cardiac fibrosis; (2) *in vitro*cell experiments lacking physiological relevance to the cardiac microenvironment; (3) Reviews with excessive repetition or low academic quality; (4) Conference abstracts, case reports, letters, and studies with incomplete data; (5) Literature published before 2018 to prioritize recent advancements.

## 3 Clinical cardiological significance of cardiac fibrosis mechanisms

Cardiac fibrosis is more than just a structural issue; it is a fundamental pathophysiological factor directly associated with significant adverse cardiovascular events (14). Firstly, fibrosis leads to severe electrical remodeling by disrupting gap junction distribution, prolonging action potential duration, and causing heterogeneous conduction slowing. These changes significantly elevate the risk of ventricular tachycardia, ventricular fibrillation, and sudden cardiac death (15). Secondly, after a myocardial infarction, scar tissue creates an abnormal boundary zone between healthy myocardium and fibrous tissue (16). This zone continuously generates pro-arrhythmic triggers, increasing susceptibility to recurrent arrhythmias (17). Thirdly, diffuse fibrosis raises myocardial stiffness, impairs both diastolic and systolic function, and contributes to the progression of heart failure, whether with reduced or preserved ejection fraction (18). Additionally, perivascular fibrosis compresses microvessels, reduces coronary flow reserve, and worsens ischemia, creating a vicious cycle of ischemia-fibrosis-ischemia (19). Consequently, understanding intercellular communication mechanisms is crucial for clinical risk stratification, early identification of high-risk patients, and the development of anti-arrhythmic and anti-remodeling therapies.

## 4 Modes and molecular mediators of intercellular communication in cardiac fibrosis

### 4.1 Paracrine and autocrine communication mediated by soluble factors

**Figure 1:**
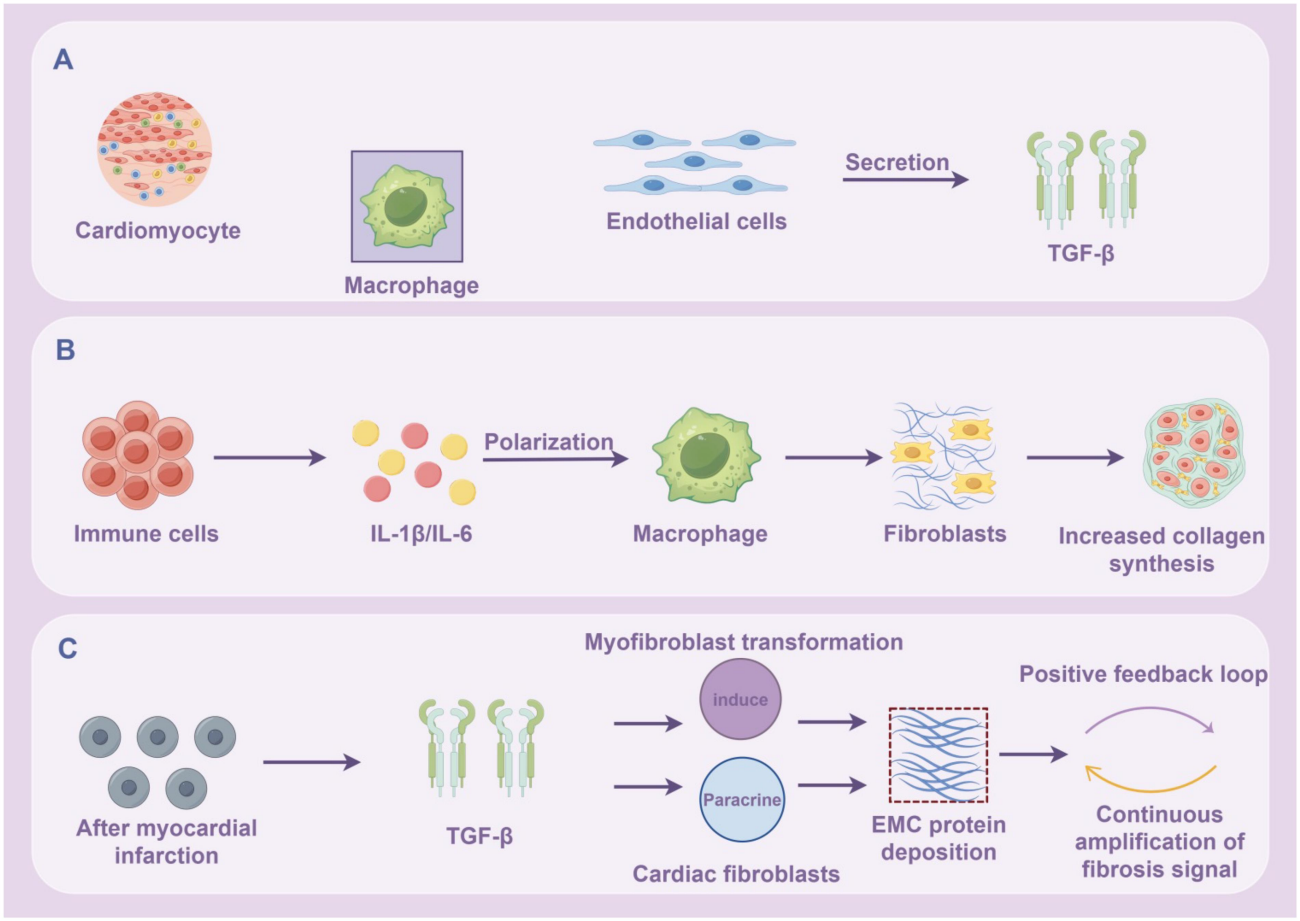
**(A)** Cardiomyocytes, macrophages, and endothelial cells produce transforming growth factor-β (TGF-β). **(B)** Immune cells release inflammatory cytokines, interleukin (IL)-1β and IL-6, which recruit and polarize macrophages. These macrophages, in turn, stimulate fibroblast proliferation and collagen synthesis. **(C)** Following a myocardial infarction, activated TGF-β engages cardiac fibroblasts via paracrine signaling, converting them into myofibroblasts and enhancing the synthesis and deposition of extracellular matrix (ECM) proteins. Additionally, TGF-β creates a positive feedback loop that continually amplifies fibrotic signaling.

Intercellular communication during myocardial fibrosis primarily relies on the paracrine and autocrine actions of soluble factors. Central to this process is the transforming growth factor-β (TGF-β) signaling pathway. TGF-β, a classic driver of fibroblast activation, is a key signaling molecule secreted by cardiomyocytes, endothelial cells, and macrophages (20). After myocardial infarction, TGF-β activates and influences cardiac fibroblasts through paracrine mechanisms, transforming them into myofibroblasts. This transformation promotes the synthesis and deposition of extracellular matrix (ECM) proteins (21). Furthermore, TGF-β can establish a positive feedback loop, continuously amplifying fibrotic signals. The ultimate result of this pathway activation is the formation of a dense scar in the infarcted region. While this reduces the risk of cardiac rupture, it also leads to myocardial stiffening, diastolic dysfunction, and progressive heart failure in the chronic phase. Beyond TGF-β, a complex network of cytokines and chemokines significantly contributes to myocardial fibrosis. In conditions such as dilated cardiomyopathy (DCM) and myocardial infarction (MI), macrophages secrete various pro-fibrotic molecules (10). Inflammatory cytokines like interleukin (IL)-1β and IL-6, primarily released by immune cells and stressed cardiomyocytes, not only recruit and polarize macrophages but also directly stimulate fibroblast proliferation and collagen synthesis (1). These cytokines induce persistent inflammatory infiltration and fibroblast activation, leading to diffuse myocardial fibrosis, ventricular wall thinning, and impaired contractility, which are characteristic pathological features of end-stage dilated cardiomyopathy (Figure 1).

The pericardial space is a crucial extra-cardiac source of soluble mediators that play a significant role in fibrotic intercellular communication (22). After myocardial injury, pericardial fluid accumulates high levels of transforming growth factor-β (TGF-β), platelet-derived growth factor (PDGF), fibroblast growth factor (FGF), tumor necrosis factor-α (TNF-α), and various chemokines such as CCL2 and CXCL8 (23). These mediators from the pericardium quickly infiltrate the myocardial matrix, triggering paracrine signaling cascades across multiple cell types (24). For instance, pericardial TGF-β and PDGF work together to promote the transition of fibroblasts to myofibroblasts, while pericardial chemokines boost the recruitment and activation of immune cells (25). Consequently, the pericardial signaling compartment functions as an acute “signaling hub,” enhancing intercellular communication and accelerating the development of cardiac fibrosis (26).

Vasoactive substances such as angiotensin II and endothelin-1, produced by endothelial cells and cardiomyocytes, activate fibroblasts through paracrine mechanisms (27). Concurrently, platelet-derived growth factor (PDGF) and fibroblast growth factor (FGF) create a complex signaling network. Single-cell RNA sequencing studies reveal that in diabetic cardiomyopathy, the interaction between PDGF ligands and their receptor PDGFRα is vital for intercellular communication, fostering a pro-fibrotic microenvironment (5). This signaling pathway specifically mediates perivascular fibrosis, reduces microvascular perfusion, and worsens myocardial ischemia, forming an ischemia-fibrosis-ischemia cycle that accelerates the progression of diabetic cardiomyopathy. Collectively, these soluble factors regulate the onset and advancement of myocardial fibrosis.

### 4.2 Extracellular vesicle-mediated long-range and local signaling

Extracellular vesicles (EVs), including exosomes and microvesicles, are vital for both long-range and local intercellular communication. These vesicles are highly diverse, with their functions largely determined by the origin of the secreting cells and the specific “cargo” they carry. The miRNAs, lncRNAs, proteins, and lipids loaded into EVs from various cell types, such as cardiomyocytes, endothelial cells, and stem cells, exhibit cell-specificity, influencing their pro-fibrotic or anti-fibrotic roles (4). In heart failure, cardiomyocytes selectively accumulate and secrete miR-30d, predominantly found in EVs (28). When these vesicles are absorbed by cardiac myofibroblasts, miR-30d inhibits their proliferation and activation through paracrine mechanisms by targeting integrin α5, thereby exerting an anti-fibrotic effect during the acute phase (28). *In vivo* experiments confirm that miR-30d overexpression significantly reduces myocardial collagen volume fraction, improves myocardial compliance, and restores left ventricular diastolic function in heart failure models. Conversely, other miRNAs may have pro-fibrotic properties. Research indicates that silencing the harmful miR-192-5p and miR-432-5p in small extracellular vesicles (sEVs) derived from cardiac c-kit + cells enhances the therapeutic potential of sEVs, reducing fibrosis and inflammatory responses after myocardial infarction (29). Modified sEVs reduce infarct size by over 30%, decrease TGF-β and CTGF expression by more than 50%, and significantly improve cardiac ejection fraction and survival in animal models. Beyond non-coding RNAs, EVs can also transport bioactive proteins and enzymes. For instance, they can deliver TGF-β, matrix metalloproteinases (MMPs), and their tissue inhibitors (TIMPs), directly modifying the microenvironment of recipient cells and affecting their function (4). The imbalance of MMPs/TIMPs transported by EVs causes ECM degradation disorder, excessive collagen deposition, and myocardial scarring, which are key drivers of post-infarction cardiac remodeling. Following myocardial infarction, EV-mediated communication is involved in various pathophysiological processes, including angiogenesis, inflammation, and fibrosis (4). Thus, extracellular vesicles, as complex signaling platforms, play multifaceted roles in regulating myocardial fibrosis through their diverse cargo.

### 4.3 Direct cell-cell contact and extracellular matrix-mediated mechanochemical signal integration

**Figure 2:**
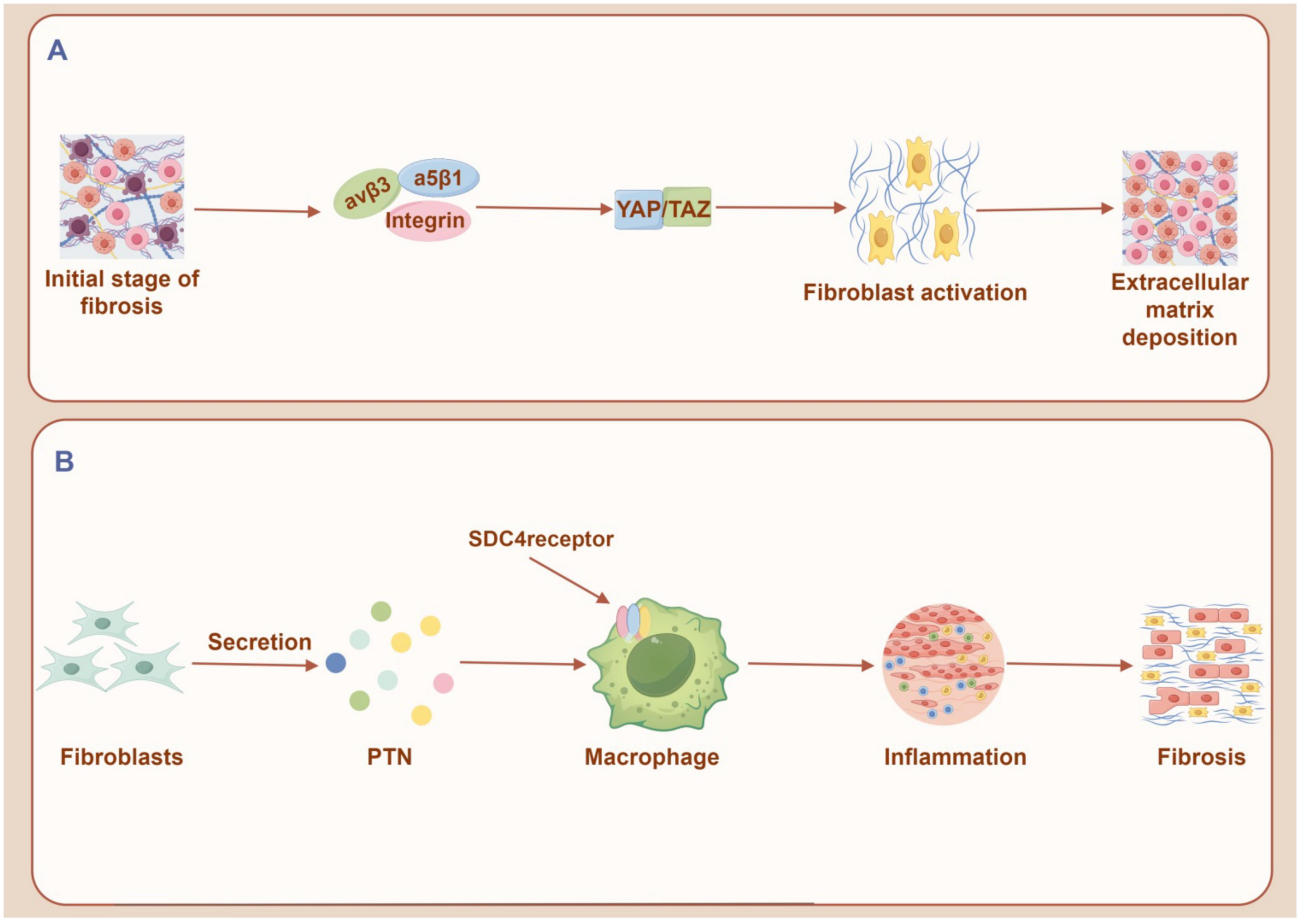
**(A)** In the early stages of fibrosis, increased stiffness of the extracellular matrix (ECM) converts mechanical signals into intracellular chemical signals via integrins αvβ3 and α5β1. This conversion activates the YAP/TAZ pathway, resulting in fibroblast activation and heightened ECM deposition. **(B)** In a mouse model of pressure-overload-induced hypertrophic cardiomyopathy, fibroblast-secreted protein PTN initiates inflammatory responses and fibrosis by binding to the SDC4 receptor on macrophages.

In myocardial fibrosis, intercellular communication occurs through soluble molecules, vesicles, direct cell-cell contact, and mechanochemical signal integration via the extracellular matrix (ECM). Gap junctions and tunnel nanotubes (TNTs) are crucial for direct communication between cells. Cardiomyocytes and fibroblasts exchange ions and small-molecule metabolites through the gap junction protein Cx43, essential for maintaining electrophysiological stability. Disruptions in these junctions can interfere with the transmission of fibrogenic signals (4). When Cx43 is downregulated or structurally abnormal, it disrupts electrical coupling between cardiomyocytes and fibroblasts, leading to reentrant arrhythmias and an increased risk of sudden cardiac death in fibrotic hearts. Conversely, TNTs facilitate the direct transfer of organelles and macromolecules between cells, offering cardioprotective effects (4). They enable the transfer of intact mitochondria from endothelial cells to injured cardiomyocytes, enhancing mitochondrial function, reducing oxidative stress, and inhibiting fibroblast activation as part of an endogenous repair mechanism. The ECM is more than a static structural scaffold; it acts as a dynamic signaling hub. In the early stages of fibrosis, increased ECM stiffness translates mechanical signals into intracellular chemical signals via integrins αvβ3 and α5β1, activating the YAP/TAZ pathway. This activation promotes fibroblast activation and further matrix deposition, creating a self-reinforcing cycle (8). Research indicates that TGF-β1 and the mechanosensitive transcription factor YAP work synergistically in 3D models of human cardiac fibrosis, advancing myofibroblast activation and matrix stiffening (8). This synergy makes fibrosis irreversible; even after the initial stimulus is removed, fibrosis continues to progress, leading to chronic heart failure. Moreover, direct contact between immune cells and matrix cells precisely regulates the fibrotic process. For example, T cells, macrophages, and fibroblasts form immunosynapses via surface adhesion molecules ICAM-1/LFA-1 to transmit activating or inhibitory signals (4). In a mouse model of stress-induced hypertrophic cardiomyopathy, interactions between cardiac fibroblasts and macrophages are common. The fibroblast-secreted protein PTN promotes fibrosis and inflammatory responses by binding to the macrophage receptor SDC4 (6). This interaction sustains macrophage M1 polarization, increases pro-inflammatory factor secretion, and triggers progressive myocardial fibrosis and hypertrophy, ultimately leading to diastolic dysfunction (Figure 2).

## 5 Roles and interactions of intracardiac cell types in communication networks

### 5.1 Classification of cell subtypes and research models in cardiac fibrosis

This section aims to clarify the intricate intercellular networks by discussing individual cellular subtypes separately. It also classifies the commonly used research models in this field, including *in vitro*cellular models, animal models, and human translational models.

#### 5.1.1 Cardiac fibroblasts

Cardiac fibroblasts are key cells responsible for maintaining extracellular matrix (ECM) balance during both heart development and pathological fibrosis (30). In embryonic and postnatal heart development, these fibroblasts are mostly inactive but proliferate moderately, secreting collagen, proteoglycans, and glycoproteins to form a structured interstitial scaffold (31). This matrix is crucial for maintaining myocardial integrity, tissue stiffness, and electrical stability, thereby supporting normal heart muscle contraction and electrical signaling between cells (32). In response to acute cardiac injury, cardiac fibroblasts act as first responders. They detect mechanical stress, inflammation, and paracrine signals to initiate tissue repair, which helps limit structural damage and preserve heart function (33). However, chronic pathological conditions such as inflammation, ischemia, neurohumoral activation, and disrupted cell communication lead to prolonged fibroblast activation. These fibroblasts then transform into myofibroblasts, marked by high α-smooth muscle actin (α-SMA) levels. These myofibroblasts deposit large amounts of collagen I, collagen III, and other ECM components, resulting in excessive matrix buildup, increased myocardial stiffness, diastolic dysfunction, and electrical remodeling (34). Additionally, activated cardiac fibroblasts release cytokines, chemokines, and growth factors that influence immune cell infiltration, endothelial cell behavior, and cardiomyocyte survival. This creates feedback loops that intensify and sustain fibrosis. Therefore, although cardiac fibrosis involves complex cellular interactions, cardiac fibroblasts are the primary agents driving ECM remodeling during both heart development and disease progression (35).

#### 5.1.2 Cardiomyocyte subtypes and functional phenotypes

**Figure 3:**
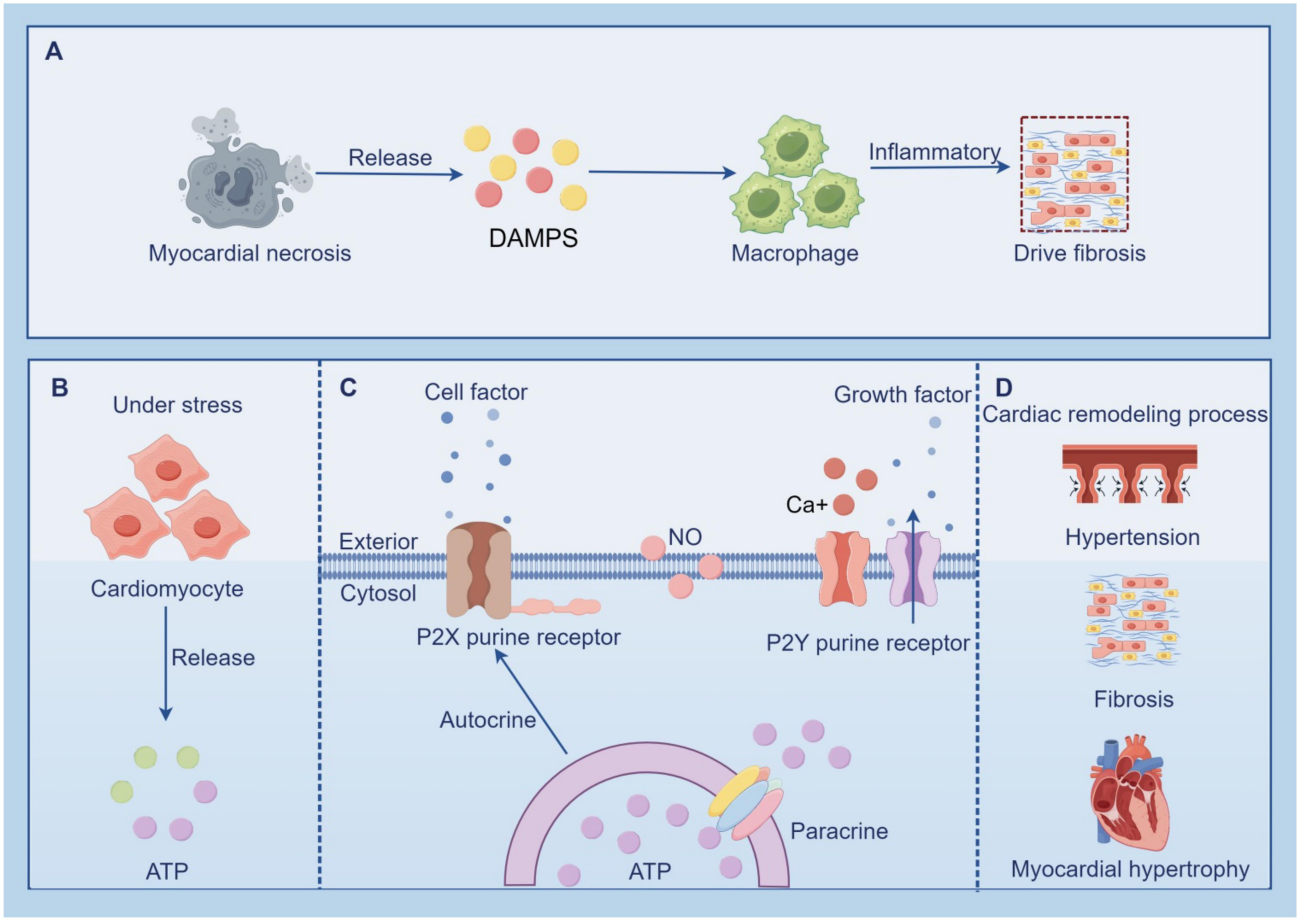
**(A)** Necrotic apoptosis in cardiomyocytes results in the release of significant amounts of DAMPs, which activate the inflammatory response in adjacent macrophages and subsequently drive fibrosis. **(B)** Under stress conditions, cardiomyocytes release DAMPs, such as adenosine triphosphate (ATP), which play a crucial role in intercellular communication. **(C)** ATP interacts with P2X and P2Y purinergic receptors, regulating calcium ions, growth factors, cytokines, and nitric oxide through both autocrine and paracrine mechanisms. **(D)** The cardiac remodeling process encompasses hypertension, fibrosis, and myocardial hypertrophy.

Cardiomyocytes, beyond their structural role, function as stress sensors and key initiators in the fibrotic process. Although they do not directly produce the extracellular matrix, they release danger signals and paracrine factors that activate the downstream fibrotic cascade. In the early stages of cardiac fibrosis, cardiomyocytes act as both initiators and amplifiers of stress signals. When exposed to mechanical stress, ischemia, or metabolic stress, these cells release signaling molecules that initiate the fibrosis network. In diabetic cardiomyopathy, cardiomyocytes create a pro-fibrotic environment by releasing the Efemp1-Egfr ligand-receptor pair (5). This pair specifically activates cardiac fibroblasts and endothelial cells, leading to perivascular and interstitial fibrosis, reduced myocardial perfusion, and impaired contractile function. Furthermore, cardiomyocytes are a major source of extracellular vesicles (EVs), which are crucial in angiogenesis, inflammation, and fibrosis after myocardial infarction (4). Under stress, cardiomyocytes release damage-associated molecular patterns (DAMPs) like adenosine triphosphate (ATP), essential for intercellular communication. ATP interacts with P2X and P2Y purinergic receptors to regulate calcium ions, growth factors, cytokines, and nitric oxide production through autocrine or paracrine mechanisms. This regulation is critical in cardiac remodeling processes, including hypertension, reperfusion injury, fibrosis, and hypertrophy (36). Excessive ATP release activates the P2X7/NLRP3 inflammasome pathway, leading to cardiomyocyte pyroptosis, inflammatory storms, fibroblast activation, and ECM deposition, creating a persistent pro-fibrotic environment. Programmed cell death in cardiomyocytes, particularly necrotic apoptosis, results in the release of substantial DAMPs. These molecules activate inflammatory responses in nearby macrophages, further advancing fibrosis. Extensive cardiomyocyte death and DAMP release lead to significant inflammatory infiltration, robust fibroblast activation, and scar formation, culminating in permanent cardiac dysfunction (Figure 3).

Abnormal electro-mechanical coupling in cardiomyocytes is essential for signal transduction. Defects in calcium handling can affect fibroblasts via gap junctions, prompting their proliferation and transformation into a secretory phenotype. This transformation contributes to the development of a fibrotic network (4). Abnormal calcium handling leads to persistent Ca2 + leakage, which activates fibroblast Ca2 + signaling, promotes the transition to myofibroblasts, and establishes a Ca2 + disorder-fibrosis-electromechanical dysfunction positive feedback loop. This loop further exacerbates cardiac impairment.

### 5.2 Immune cells (macrophages, lymphocytes): the regulatory hub of inflammation and repair

#### 5.2.1 Immune cell subtypes

Immune cells are categorized into macrophage subtypes and lymphocyte subsets, each playing distinct roles in either promoting or inhibiting fibrosis.

##### 5.2.1.1 Macrophage subsets

Macrophages serve as the central regulatory hub in the immune system, determining whether inflammation resolves or progresses to chronic fibrosis. They exhibit diverse functions: M1 macrophages drive inflammation, while M2 macrophages promote matrix deposition. Their plasticity and heterogeneity significantly influence fibrosis outcomes through phenotypic polarization and dynamic interactions. Cardiac macrophages are divided into two distinct populations: resident macrophages, originating from embryonic yolk sac or fetal liver progenitors, which self-renew locally under normal conditions, and recruited macrophages, derived from circulating monocytes that infiltrate the heart during tissue injury or stress. In conditions such as dilated cardiomyopathy (DCM), ischemic cardiomyopathy (ICM), myocardial infarction (MI), and heart failure (HF), macrophages contribute to myocardial fibrosis by secreting pro-fibrotic molecules (10). During the early, acute phase, resident macrophages dominate, initiating rapid immune surveillance and mild repair responses. In contrast, during the subacute and chronic phases, circulating monocyte-derived macrophages become predominant, driving sustained inflammation and progressive fibrosis. Initially, pro-inflammatory M1 macrophages release tumor necrosis factor-α (TNF-α) and interleukin-1β (IL-1β), exacerbating tissue damage. Excessive activation of M1 macrophages leads to severe myocardial inflammation, cardiomyocyte apoptosis, and an imbalance in extracellular matrix (ECM) degradation, resulting in acute ventricular dilation and dysfunction. Subsequently, reparative M2 macrophages facilitate fibroblast activation and ECM deposition by secreting transforming growth factor-β (TGF-β), platelet-derived growth factor (PDGF), and IL-10, while also modulating T-cell responses (10). However, excessive M2 polarization can lead to exaggerated ECM deposition and scar hyperplasia, reducing myocardial elasticity and causing chronic diastolic dysfunction. Single-cell transcriptomic analysis has identified macrophage-to-myofibroblast transdifferentiation (MMT) as a potential additional source of fibrosis (10). Notably, MMT predominantly occurs in monocyte-derived recruited macrophages rather than resident macrophages, underscoring the distinct roles of different macrophage origins. MMT significantly increases myofibroblast numbers in fibrotic lesions, enhances collagen synthesis, and accelerates fibrosis progression, representing a key target for anti-fibrotic therapies.

##### 5.2.1.2 T-cell subsets

T cells, including Th1, Th2, Th17, and Treg subsets, play a pivotal role in regulating fibrosis through distinct cytokine secretion patterns. Th1 cells, for example, secrete interferon-*γ* (IFN-*γ*), which has anti-fibrotic properties. In contrast, Th2 cells secrete IL-4 and IL-13, and Th17 cells secrete IL-17, both of which primarily promote fibrosis (37). Regulatory T cells (Tregs) help protect against fibrosis by suppressing immune responses and secreting the anti-fibrotic factor IL-10 (38, 39). In heart failure, the expansion of Tregs is associated with cardiac function recovery, and their immune regulation is crucial for the enhancement of diastolic function through cell therapy. Similar to macrophages, T lymphocytes consist of tissue-resident memory T cells and circulating naive/effector T cells. These cells are recruited to the injured myocardium via endothelial adhesion molecules and chemokine gradients. A deficiency in Tregs can lead to uncontrolled inflammation and excessive fibroblast activation, worsening fibrosis. Conversely, Treg supplementation can reduce inflammatory infiltration by over 60% and significantly improve cardiac function. Additionally, circulating monocytes are recruited to the heart and differentiate into macrophages. Chemokines and chemoattractants derived from pericardial fluid further enhance monocyte recruitment and macrophage infiltration, creating a positive feedback loop that exacerbates inflammatory and fibrotic responses. The interactions between these macrophages and resident cardiac fibroblasts collectively influence fibrosis outcomes (40, 41). In stress-induced hypertrophic cardiomyopathy, pluripotent growth factor (PTN) secreted by cardiac fibroblasts stimulates macrophage inflammatory responses via the multidrug-resistant glycoprotein 4 (SDC4) receptor, promoting myocardial fibrosis (6). This interaction forms a positive feedback loop: fibroblast activation leads to macrophage inflammation, which in turn causes further fibroblast activation, driving progressive hypertrophy and fibrosis.

### 5.3 Endothelial cells and pericytes

#### 5.3.1 Vascular cell subtypes

Endothelial cells and pericytes form the vascular-stromal interface, playing a crucial role in microvascular-related fibrosis by expanding the myofibroblast pool through EndMT and pericyte detachment. These cells are integral to the cellular communication network in cardiac fibrosis (42, 43). Beyond serving as a vascular barrier, endothelial cells act as active signaling centers. When stimulated by TGF-β, they undergo endothelial-mesenchymal transition (EndMT), transforming into secretory mesenchymal cells that significantly impact the myofibroblast pool and extracellular matrix (44). EndMT leads to endothelial barrier loss, microvascular rarefaction, and an increased myofibroblast supply, contributing to myocardial ischemia and fibrosis—a key mechanism of microvascular dysfunction in fibrotic hearts. In conditions like diabetic cardiomyopathy, endothelial cell numbers decline, and changes in their gene expression and chromatin accessibility affect contraction- and growth-related pathways, indicating endothelial injury and functional impairment (45). This injury suppresses angiogenesis, impairs nutrient and oxygen supply, exacerbates cardiomyocyte damage, and activates fibroblasts, creating a vicious cycle of vascular injury, fibrosis, and vascular deterioration. Activated endothelial cells promote immune cell extravasation by expressing adhesion molecules and secreting vascular endothelial growth factor (VEGF) and angiopoietin-2, which influence vascular stability and paracrine signaling (46, 47). For example, in diabetic cardiomyopathy, analyses of fibroblast-centered communication have revealed interactions between fibroblasts and endothelial cells involving VEGF receptors (45). Pericytes, which envelop microvessels, are another potential source of myofibroblasts (48, 49). During cardiac fibrosis progression, pericytes become activated and detach from the vascular wall, a process regulated by endothelial cells and the local microenvironment (50). A bioartificial cardiac tissue model using pericyte-like cells from human pluripotent stem cells demonstrated that pericytes significantly influence stromal collagen deposition and cardiac tissue stiffening, underscoring their dual role in vascularization and fibrosis-related cardiac tissue remodeling (50). Pericyte detachment induces microvascular instability and perivascular fibrosis, reducing myocardial perfusion and accelerating cardiac dysfunction. Additionally, cell communication network factor 5 (CCN5), primarily expressed in endothelial cells, fibroblasts, and macrophages, is induced in the late stages of scar healing after myocardial infarction. It suppresses myocardial collagen deposition by antagonizing the pro-fibrotic CCN2 (51). CCN5 reduces scar area by inhibiting excessive ECM deposition, improves myocardial elasticity, and preserves post-infarction cardiac function, representing a promising endogenous anti-fibrotic factor. These findings highlight the central role of vascular unit cells in sensing microenvironmental changes, transmitting mechanobiological signals, and directly participating in fibrosis formation.

### 5.4 Overview of research models in intercellular communication studies

To enhance understanding and comparison, commonly used models in cardiac fibrosis intercellular communication research are categorized as follows:

#### 5.4.1 *In vitro* models

The monoculture model is employed to investigate the behavior of individual cells. In contrast, the co-culture model simulates direct communication between cells. Meanwhile, the 3D cardiac tissue model is utilized to replicate extracellular matrix (ECM) stiffness and the mechanical microenvironment.

#### 5.4.2 Animal models

The mouse model includes pressure overload through transverse aortic constriction (TAC), myocardial infarction (MI), and isoproterenol (ISO) infusion. The rat model is used for studying volume overload and diabetic cardiomyopathy. The porcine model is particularly valuable due to its close resemblance to human cardiac anatomy and electrophysiology.

#### 5.4.3 Human-derived models

Human stem cell-derived cardiomyocytes and fibroblasts, along with human myocardial tissue samples, provide valuable insights into cardiac biology. Additionally, single-cell sequencing data from patients offer a detailed understanding of cellular heterogeneity and function.

## 6 Intercellular communication axes driving pathological fibrosis

### 6.1 The PDGF-PDGFR axis

The axis in question is not merely “important” but stands as the most powerful signal for fibroblast proliferation and migration among all paracrine pathways involved in cardiac fibrosis. The platelet-derived growth factor (PDGF) and its receptor (PDGFR) signaling pathway are pivotal in tissue fibrosis, significantly impacting fibrotic processes across various organs (52). This pathway triggers receptor dimerization and autophosphorylation through ligand-receptor binding, activating downstream intracellular signaling cascades. These cascades facilitate fibroblast proliferation, migration, and extracellular matrix deposition (52). In myocardial fibrosis, single-cell studies have pinpointed the interaction between PDGF ligands and PDGFRα on fibroblasts as a crucial signaling axis driving fibrosis (53, 54). In myocardial ischemia and reperfusion injury (MI/RI) models, M2b macrophages have been shown to significantly mitigate myocardial remodeling and fibrosis by modulating PDGFR kinase activation in cardiac fibroblasts (55). *In vivo* experiments demonstrate that inhibiting the PDGF-PDGFRα axis reduces myocardial collagen volume fraction by over 40%, enhances ventricular wall stiffness, and restores ejection fraction to nearly normal levels. This evidence underscores the potential of targeting the PDGFRα axis as a therapeutic strategy. Notably, PDGF signaling in fibrosis is cell-type specific. This specificity implies that targeting ligand-receptor interactions between particular cell types, rather than broadly inhibiting a single molecule, could offer more precise treatment options with fewer side effects. Broad PDGF inhibition might impair angiogenesis and delay wound healing, whereas cell-specific targeting avoids these adverse effects and enhances therapeutic safety.

### 6.2 The Spp1-Itgb1 axis

This axis functions as a specialized molecular bridge, linking immune cell activation to fibroblast transformation, rather than serving as a general pathway. Osteopontin (OPN), encoded by the Spp1 gene, is crucial in connecting immune responses to the fibrotic process, significantly influencing the intercellular communication network in cardiac fibrosis (56, 57). OPN is primarily expressed by macrophages (58), and its receptors include integrin family members αvβ1, αvβ3, αvβ5, and CD44. In pathological conditions, the interaction between myeloid-derived OPN and cardiac fibroblasts is vital for pro-fibrotic signaling (59, 60). Research highlights integrin β1 (Itgb1) as a key gene in fibroblasts, facilitating intercellular signaling in various fibrotic diseases (61). While this study focuses on the PDGF pathway, it reveals a complex communication network among immune cells, endothelial cells, and fibrogenic effector cells through specific ligand-receptor interactions. In the myocardium, the Spp1-Itgb1 axis establishes a communication link between macrophages and fibroblasts. Itgb1, highly expressed in fibroblasts, integrates OPN signals from immune cells, activating downstream pro-fibrotic pathways. Activation of this axis upregulates TGF-β, CTGF, and other pro-fibrotic factors, promoting fibroblast transition and collagen synthesis, leading to diffuse myocardial fibrosis and cardiac dysfunction. Related studies suggest using adeno-associated virus (AAV)-mediated cardiac-specific Itgb1 knockout as a potential intervention to improve fibrosis. Cardiac-specific Itgb1 knockout significantly reduces OPN-induced fibroblast activation and ECM deposition, enhances myocardial compliance, and effectively reverses myocardial fibrosis in animal models. This strategy indicates that targeting the Spp1-Itgb1 axis to disrupt pathological intercellular communication can alleviate myocardial fibrosis (62).

## 7 The neuro-immune-cardiac axis

### 7.1 Excessive activation of the sympathetic nervous system

**Figure 4:**
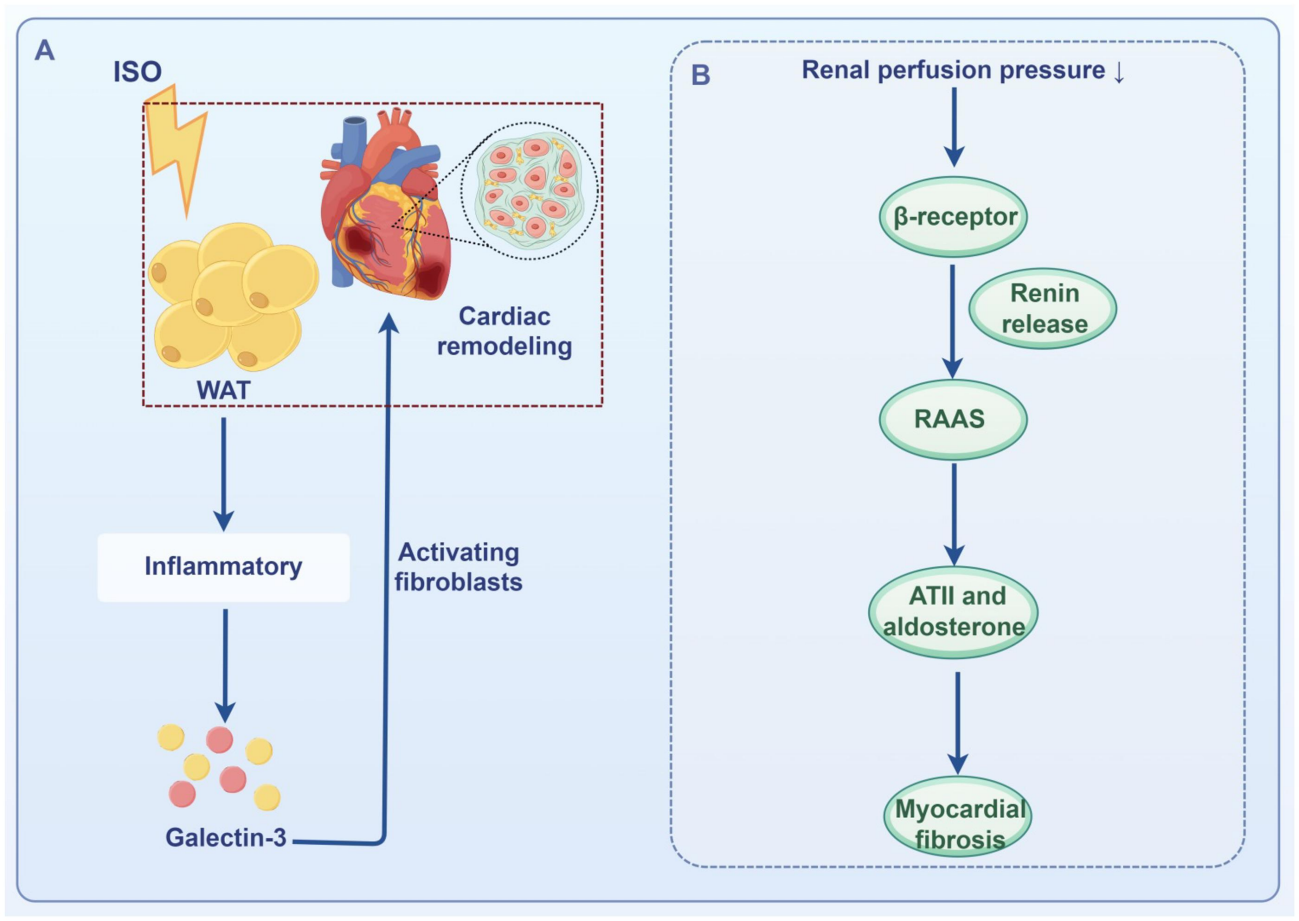
**(A)** Isoproterenol (ISO) induces inflammation in white adipose tissue (WAT), which leads to the secretion of galectin-3. This secretion, in turn, activates cardiac fibroblasts and promotes cardiac remodeling. **(B)** A decrease in renal perfusion pressure activates the β-adrenergic receptors of the sympathetic nervous system, resulting in renin release and subsequent activation of the RAAS system. This process encourages myocardial hypertrophy and fibrosis through the actions of angiotensin II (AT-II) and aldosterone.

Sympathetic hyperactivation is not merely a contributing factor but a sustained neurohumoral driver that perpetuates and intensifies fibrosis long after the initial injury. In chronic heart failure, persistent overactivation of the sympathetic nervous system (SNS) is a crucial pathophysiological mechanism that accelerates myocardial fibrosis. This heightened sympathetic activity leads to increased norepinephrine (NE) release, which interacts with adrenergic receptors on fibroblasts, cardiomyocytes, and immune cells. This interaction promotes collagen synthesis and inflammatory responses, creating a self-sustaining cycle of fibrosis (63). For example, following a myocardial infarction (MI), stressors such as light-induced stress can exacerbate cardiac sympathetic remodeling. This results in excessive activation of the brain-heart SNS, further impairing cardiac function and increasing fibrosis (64). Sympathetic overactivation elevates the myocardial collagen I/III ratio by 2–3-fold, induces stiffening and diastolic dysfunction, and significantly reduces survival in heart failure models. The SNS overactivation affects the heart both directly and indirectly by influencing adipose tissue. Research shows that chronic β-adrenergic stimulation, such as with isoproterenol (ISO), induces inflammation in white adipose tissue (WAT) and triggers the secretion of galectin-3. Galectin-3 is a potent fibrogenic agent that activates cardiac fibroblasts, thereby promoting cardiac remodeling (65). Adipose-derived galectin-3 acts as an endocrine factor, remotely activating cardiac fibroblasts and triggering systemic inflammation and myocardial fibrosis, a key mechanism in obesity-related cardiomyopathy. Moreover, sympathetic activation is closely linked with the renin-angiotensin-aldosterone system (RAAS), which together drive the fibrotic process. Sympathetic β-adrenergic receptors respond to decreased renal perfusion pressure by triggering renin release and activating the RAAS. In turn, angiotensin II (AT-II) and aldosterone contribute to myocardial hypertrophy and fibrosis (66). The synergy between the SNS and RAAS forms a powerful pro-fibrotic network, causing severe myocardial hypertrophy and fibrosis, the core pathological basis of hypertensive heart disease and chronic heart failure (Figure 4).

In heart failure with preserved ejection fraction (HFpEF), excessive sympathetic activation forms a complex pathological network that involves inflammation and metabolic stress. This network increases myocardial ATP levels, activating the P2X7/NLRP3 inflammasome axis, which leads to oxidative stress, pyroptosis, and interstitial fibrosis (67). Leptin significantly contributes to the onset and progression of HFpEF, with its elevated levels closely associated with sympathetic nervous system activation, further promoting myocardial fibrosis (68). Intervention strategies targeting this excessive sympathetic activation have shown therapeutic promise. For example, renal denervation (RDN) effectively reduces systemic and myocardial norepinephrine (NE) levels. In a porcine myocardial infarction (MI) model, RDN combined with exosome treatment derived from autologous peripheral blood mesenchymal stem cells (PBMSC-ExosRD) significantly enhanced cardiac repair and reduced fibrosis. This was achieved by inhibiting the sympathetic nervous system and transferring the miR-141-200-429 cluster to ischemic cardiomyocytes (69). This combined therapy reduced fibrosis area by over 50%, improved left ventricular ejection fraction (LVEF) by more than 25%, and effectively reversed cardiac remodeling in large animals. Similarly, in a mouse model of HFpEF, RDN improved diastolic function and reduced ventricular remodeling by inhibiting the SNS-driven ATP-P2X7-NLRP3 inflammasome axis (67). Additionally, targeting triglyceride lipase (ATGL) in adipose tissue to inhibit lipolysis has been shown to improve ISO-induced cardiac remodeling by reducing adipose tissue inflammation and galectin-3 secretion (65). Melatonin therapy has also demonstrated benefits, improving cardiac remodeling and mitigating brain-heart sympathetic hyperactivation exacerbated by light disruption following MI (64). Overall, these studies highlight the central role of sympathetic hyperactivation in myocardial fibrosis and suggest new directions for combating fibrosis through multifaceted interventions targeting the sympathetic signaling network.

Substance P and calcitonin gene-related peptide (CGRP), released by cardiac sensory neurons, are essential in regulating local cardiac immune and fibrotic responses. They interact with the sympathetic nervous system to influence the fibrotic microenvironment. CGRP, mainly produced by sensory neurons, possesses strong vasodilatory, cardioprotective, anti-inflammatory, and anti-fibrotic properties, playing a crucial role in cardiovascular homeostasis (70). In the early stages of heart failure, elevated CGRP levels help mitigate damage from sympathetic overactivation and the renin-angiotensin-aldosterone system (RAAS) by reducing afterload, maintaining endothelial integrity, and preventing ventricular remodeling (70). However, as heart failure progresses, CGRP depletion, receptor downregulation, and impaired signaling reduce its protective effects, leading to increased fibrosis and extracellular matrix disruption (70). The depletion of CGRP disrupts the balance between vasoconstriction and vasodilation, increases endothelial permeability, and enhances fibroblast activation, accelerating fibrosis progression. CGRP's antifibrotic mechanism may involve directly inhibiting fibroblast activation and collagen deposition, as well as modulating local immune responses. The release of sensory neuropeptides is regulated by inflammatory factors and mechanical stress, involving interactions with sympathetic neurotransmitters. Excessive sympathetic activation, for instance, can affect sensory neuron excitability and neuropeptide release. Therapeutic strategies targeting the CGRP signaling pathway show promise. These include CGRP agonists, the stable analog SAX, gene-based therapies to enhance endogenous CGRP expression, receptor modulators, and TRPV1-induced release stimulators (70). Preclinical and early clinical studies suggest these interventions can improve cardiac output, reduce infarct size, and protect ventricular function (70). CGRP agonists have been shown to reduce myocardial collagen deposition by 35%–40%, improve compliance, and effectively alleviate heart failure symptoms.

### 7.2 The cholinergic anti-inflammatory pathway

This pathway functions as an intrinsic protective regulator that curtails excessive inflammation-driven fibrosis, rather than merely serving as a key pathway (71). The cholinergic anti-inflammatory pathway forms a crucial connection between the autonomic nervous system and the immune system, significantly affecting myocardial fibrosis development (72). This pathway operates through the neurotransmitter acetylcholine, which is released upon vagal nerve activation. Acetylcholine binds to the α7 nicotinic acetylcholine receptor (α7nAChR) on macrophages, inhibiting the nuclear factor *κ*B signaling pathway (73). Consequently, the release of pro-inflammatory and pro-fibrotic cytokines is reduced, establishing an endogenous anti-fibrotic defense mechanism (74). Numerous studies underscore the significance of this pathway in cardiac protection. For example, in a rat model of myocardial infarction, activating the α7nAChR specifically enhances cardiac function and reduces cardiac fibrosis. This outcome is closely associated with the suppression of pro-inflammatory M1 macrophage polarization, the promotion of anti-inflammatory M2 macrophage polarization, and the regulation of macrophage phenotypes via the STAT3 signaling pathway (75). Activation of α7nAChR decreases TNF-α and IL-1β expression by over 60%, reduces collagen volume fraction by approximately 45%, and significantly improves left ventricular ejection fraction (LVEF) and survival (76).

In rats with ischemia-induced chronic heart failure, the activation of the cholinergic pathway through the drug agonist PNU-282987 significantly reduces both systemic and cardiac inflammatory responses (77). This activation helps alleviate myocardial fibrosis and improves mitochondrial dysfunction associated with excessive inflammation, thus preventing ischemic ventricular remodeling (77). PNU-282987 enhances mitochondrial membrane potential, decreases ROS production, restores ATP synthesis, and inhibits fibrosis by suppressing inflammation (78). These findings underscore the importance of the anti-inflammatory effects mediated by the α7 nicotinic acetylcholine receptor (α7nAChR) in preventing myocardial fibrosis and promoting improved cardiac remodeling.

The cholinergic anti-inflammatory pathway plays a vital role in mitigating inflammation and fibrosis, making vagus nerve activation through physical or electrical stimulation a promising therapeutic approach (79). Focused ultrasound stimulation, a non-invasive neuromodulation technique, has demonstrated significant cardioprotective effects in rat models of acute myocardial ischemia and reperfusion injury. Research shows that stimulating the right cervical vagus nerve with focused ultrasound during early reperfusion reduces arrhythmias, myocardial infarction, and fibrosis while preserving left ventricular ejection fraction. Specifically, focused ultrasound decreases infarct size by approximately 30%, reduces ventricular arrhythmia incidence by over 50%, and effectively maintains cardiac function (80). Conversely, vagotomy or the administration of the cholinergic receptor antagonist atropine negates these protective effects, underscoring the importance of the cholinergic anti-inflammatory pathway (81). Additionally, electrical stimulation of certain brain regions can activate this pathway. For example, stimulating the cerebellar vermis increases serum acetylcholine levels, decreases TNF-α and IL-6, and reduces inflammatory cell infiltration, infarct size, and fibrosis. This cardiac neuroprotective mechanism is believed to enhance vagal tone, release acetylcholine, and activate the cholinergic anti-inflammatory pathway through α7 nicotinic acetylcholine receptors (α7nAChR) (82). This central neuromodulation strategy offers a novel non-pharmacological approach for addressing myocardial fibrosis and heart failure (83). These interventions provide innovative therapeutic options for modulating the neuroimmune dialogue and managing cardiac inflammation and fibrosis.

The cholinergic anti-inflammatory pathway plays a crucial role in protecting the heart from stress-induced injury. Deficiencies in this pathway can exacerbate cardiac injury and fibrosis. Research shows that the absence of the α7 nicotinic acetylcholine receptor (α7nAChR) increases the heart's susceptibility to damage. In an isoprenaline-induced adrenergic stress model, α7nAChR knockout mice exhibited severe myocardial fibrosis, significant CCR2 + monocyte infiltration, and increased cardiomyocyte death compared to wild-type mice (84). The deletion of α7nAChR leads to uncontrolled inflammation and fibroblast activation, resulting in a threefold increase in collagen deposition and significantly reduced contractility. This indicates that α7nAChR not only offers anti-inflammatory protection but may also independently support cardiomyocyte survival. In myocardial infarction models, the lack of α7nAChR exacerbates cardiac fibrosis by activating the NF-*κ*B pathway and promoting endothelial-mesenchymal transition (85). Furthermore, in viral myocarditis models, α7nAChR on B cells functions as a negative regulator, suppressing pro-inflammatory activities and related signaling pathways. Ablating α7nAChR specifically in B cells leads to increased myocardial inflammation and fibrosis (86). These findings underscore the importance of cholinergic signaling, particularly through α7nAChR, as a vital endogenous mechanism for controlling excessive inflammatory responses and preventing myocardial fibrosis. Impairment of this function significantly contributes to disease progression.

Research on the cholinergic anti-inflammatory pathway has progressed from foundational studies to clinical applications, emphasizing both neurostimulation techniques and pharmacological interventions (87). A key pharmacological strategy involves acetylcholinesterase inhibitors like donepezil. By inhibiting acetylcholine degradation, donepezil enhances cholinergic signaling, demonstrating cardioprotective effects in rats with chronic heart failure. Notably, these benefits diminish when peripheral, rather than central, α7 nicotinic acetylcholine receptors (α7nAChRs) are blocked, highlighting the critical role of peripheral α7nAChRs in treating heart failure with cholinergic agents (88). Donepezil reduces fibrosis area by approximately 40%, improves diastolic function, and exhibits favorable safety and tolerability in preclinical studies. Similarly, in a model using spontaneously hypertensive rats, electroacupuncture at the “Neiguan” acupoint improved myocardial fibrosis (89). This effect was reversed by α7nAChR antagonists and correlated with increased acetylcholine levels in myocardial tissue (90). Additionally, there was a downregulation of pro-inflammatory factors such as NF-*κ*B, p65, TNF-α, IL-1β, and IL-6. These findings suggest that the cholinergic anti-inflammatory pathway, along with its downstream NF-*κ*B signaling, plays a significant role in the anti-fibrotic process.

### 7.3 Overview of cardiac fibrosis pathogenesis and core mechanisms

Cardiac fibrosis is a complex and progressive pathological process influenced by various stimuli and coordinated through multicellular interactions (91). It unfolds in three stages: initiation, progression, and persistent remodeling. During the initiation stage, factors such as mechanical stress, ischemia-reperfusion injury, metabolic disorders, neurohumoral activation, and inflammatory infiltration lead to cardiomyocyte damage, immune cell recruitment, and endothelial dysfunction (92). Damaged cardiomyocytes release damage-associated molecular patterns (DAMPs) and pro-inflammatory factors, which activate resident immune cells and attract circulating immune cells. In the progression stage, these activated immune cells secrete pro-fibrotic cytokines like TGF-β, PDGF, and IL-1β, prompting the transformation of quiescent cardiac fibroblasts into pro-fibrotic myofibroblasts. Concurrently, endothelial cells undergo endothelial–mesenchymal transition (EndMT), and pericytes detach from microvessels, both contributing additional sources of myofibroblasts (93). During the persistent remodeling stage, myofibroblasts continuously produce and deposit extracellular matrix (ECM) components, such as collagen I and III, which increase myocardial stiffness, impair diastolic function, and disrupt electrical signals. This abnormal ECM environment further activates mechanosensitive signaling pathways, like YAP/TAZ, through integrins, creating a self-reinforcing positive feedback loop that renders fibrosis irreversible. Throughout the entire process, intercellular communication networks—mediated by paracrine signals, extracellular vesicles, direct cell contact, and ECM–cell interactions—play a crucial role in determining the intensity, duration, and reversibility of cardiac fibrosis. A comprehensive understanding of these core mechanisms is essential for developing precise anti-fibrotic therapeutic strategies.

## 8 Intervention strategies targeting intercellular communication networks and future prospects

### 8.1 Current antifibrotic therapies: clinical applications, efficacy and limitations

Current clinical antifibrotic therapies primarily focus on indirect strategies that target neurohumoral regulation and symptom improvement (94). While these approaches have shown some success, they also present significant limitations (95). First, RAAS inhibitors, such as ACEI/ARB and MRA, are first-line treatments that reduce angiotensin II and aldosterone levels to inhibit myocardial fibrosis. They improve myocardial remodeling and cardiac function but cannot reverse established fibrosis and are less effective in non-ischemic cardiomyopathy (96). Second, SGLT2 inhibitors are newly recommended for their confirmed antifibrotic effects, which reduce myocardial stiffness and improve heart failure prognosis. However, their mechanism is indirect and does not specifically address intercellular communication disorders (97). Third, Pirfenidone, an antifibrotic drug used for pulmonary fibrosis, has shown potential in preclinical studies for myocardial fibrosis. Despite this promise, it lacks sufficient clinical evidence and has systemic side effects (98). Fourth, interventional and device therapies, such as renal denervation, can address sympathetic overactivation-related fibrosis but suffer from poor applicability and individual variability. The main limitations of these therapies include a lack of cell specificity, inability to target core intercellular communication networks, difficulty in reversing established fibrosis, significant side effects with long-term use, and poor efficacy in diverse patient populations (99). These challenges highlight the urgent need for new, precise antifibrotic strategies that specifically target intercellular communication networks.

### 8.2 Targeting key communication molecules and signaling pathways

Targeting core signaling molecules and pathways in intercellular communication is a widely researched anti-fibrotic strategy, aiming to disrupt pro-fibrotic signal transmission and demonstrating significant preclinical success (100). The TGF-β/Smad axis is the primary pro-fibrotic pathway, facilitating the transition from fibroblasts to myofibroblasts and working with YAP to enhance fibrotic responses (101). Current targeted treatments include small-molecule inhibitors like galunisertib and pirfenidone, neutralizing antibodies, and Smad3-targeted gene therapy tools. Preclinical studies indicate that galunisertib can reduce myocardial collagen deposition by 50%–60% and improve tissue stiffness (102). However, prolonged systemic use can lead to immunosuppression and delayed wound healing, hindering its clinical application. Beyond TGF-β, the PDGF-PDGFRα, Spp1-Itgb1, and PTN-SDC4 pathways are also critical targets for intervention (103). PDGFRα inhibitors specifically inhibit fibroblast proliferation and migration, with ongoing validation in large animal models for cardiac fibrosis (104). In the realm of inflammatory signaling, monoclonal antibodies targeting IL-11, CCL2, and TNF-α significantly reduce inflammatory infiltration and fibroblast activation by over 50%, with high specificity and minimal off-target effects. Non-coding RNA therapy is another promising avenue: miR-29 and miR-30d mimics, along with anti-miR-21, directly regulate collagen and pro-fibrotic factor expression. Engineered vector delivery enhances targeting and stability, making these nucleic acid drugs highly promising.

### 8.3 Regulation of extracellular vesicle biogenesis, secretion, and uptake

Extracellular vesicles (EVs) play a crucial role in intercellular communication. Regulating their biogenesis, secretion, and uptake can block pathological signal transmission and facilitate therapeutic molecule delivery, offering significant translational potential (105). Pathological EVs from cardiomyocytes, macrophages, and endothelial cells carry pro-fibrotic miRNAs and proteins that activate fibroblasts and promote fibrosis (106). Conversely, engineered therapeutic EVs can be loaded with anti-fibrotic molecules for targeted intervention (107). Current strategies for EV intervention fall into three main categories: 1. **Inhibiting Pathological EV Biogenesis and Secretion:** Targeting enzymes and proteins such as nSMase2, Rab GTPases, and using GW4869 can reduce pro-fibrotic EV secretion by over 70%. However, non-specific inhibition can disrupt normal vesicle signaling, necessitating targeted optimization (108). 2. **Modifying EV Cargo:** By knocking down pro-fibrotic miR-192-5p and miR-432-5p in cardiac progenitor small EVs, repair capacity is enhanced, reducing infarct size and fibrosis by more than 30%. Additionally, loading miR-29 and miR-133 into MSC-derived exosomes allows for fibrotic myocardium-specific accumulation, offering superior efficacy compared to free RNA (109). 3. **Blocking Pathological EV Uptake:** Targeting integrin-mediated internalization can interrupt the transmission of pro-fibrotic signals. Engineered EVs demonstrate excellent biocompatibility and low immunogenicity, making them a promising next-generation targeted delivery system for treating cardiac fibrosis.

### 8.4 Cell therapy and multi-target integrated strategies

Cell therapy offers a comprehensive anti-fibrotic approach by regulating the immune microenvironment and cellular communication networks through exogenous cell transplantation or paracrine effects (110). Mesenchymal stem cells (MSCs) are extensively studied for their therapeutic potential, primarily via paracrine signaling and exosomes. These mechanisms modulate macrophage polarization, expand regulatory T cells (Tregs), and inhibit both inflammation and fibroblast activation (111). In heart failure with preserved ejection fraction (HFpEF) models, neonatal MSCs and their secretome significantly enhance diastolic function by expanding Tregs three to four times. Treg monotherapy alone can reduce inflammatory infiltration by over 60% and reverse fibrosis. Another innovative strategy involves targeting fibroblast progenitor cells, specifically Sca1 + and CD34 + cells (112). Under stress, these progenitors differentiate into myofibroblasts. Partial depletion of these cells can reduce activated fibroblasts by approximately 60% and improve cardiac function (113). Advances in single-cell and spatial transcriptomics facilitate precision cell therapy by targeting pathological cell subsets, such as Hrc-high fibroblasts and inflammatory macrophages, thereby avoiding interference with normal cells and enhancing therapeutic efficacy. Single-target therapies often fail to reverse complex fibrosis, highlighting the importance of multi-target combination strategies. For instance, combining TGF-β inhibitors with extracellular vesicle (EV) therapy or pairing sympathetic inhibition with cell transplantation can simultaneously address inflammation, fibroblast activation, and extracellular matrix (ECM) deposition, offering superior efficacy compared to monotherapies. Additionally, stage-adjusted sequential therapy—focusing on acute anti-inflammation followed by chronic anti-fibrosis—maximizes therapeutic benefits while minimizing adverse effects.

### 8.5 Emerging locally delivered targeted therapeutic strategies

Emerging locally delivered targeted therapies are revolutionizing precision anti-fibrotic interventions by addressing the limitations of systemic cell therapy, such as off-target effects and low cardiac retention. These strategies act directly on the fibrotic myocardial microenvironment, enhancing intercellular communication regulation with greater spatial specificity and reduced systemic toxicity. Biodegradable cardiac hydrogels, like gelatin methacryloyl and hyaluronic acid hydrogels, serve as localized delivery carriers. They enable the sustained release of anti-fibrotic agents, including small molecule inhibitors, neutralizing antibodies, and miRNAs, at the site of myocardial injury. In myocardial infarction models, these hydrogels, loaded with TGF-β/Smad inhibitors or miR-29 mimics, maintain long-term retention in the infarcted area. This continuous presence effectively blocks pro-fibrotic signaling among fibroblasts, immune cells, and endothelial cells, significantly reducing extracellular matrix deposition without impacting peripheral organs. Cardiac tissue-specific gene delivery via local viral vectors, such as adeno-associated virus (AAV) serotypes with cardiac tropism like AAV9, achieves precise myocardial transfection through direct intramyocardial injection or intracoronary infusion (114). This method allows for site-specific silencing or overexpression of key communication molecules. For instance, AAV-mediated local knockout of Itgb1 in cardiac fibroblasts disrupts the Spp1-Itgb1 immune-fibrotic communication axis, alleviating myocardial fibrosis without systemic immune interference (115). Similarly, local overexpression of CCN5 via AAV inhibits endothelial-mesenchymal transition and fibroblast activation in the fibrotic zone. Implantable microfabricated devices or biodegradable patches attached to the epicardium provide on-demand, controlled release of therapeutic agents. These devices can be programmed to release anti-fibrotic cytokines, EVs, or signaling pathway inhibitors in response to pathological cues, such as matrix stiffness or inflammatory signals (116). This dynamic regulation of intercellular communication in the fibrotic microenvironment is demonstrated in preclinical models of chronic myocardial fibrosis, where epicardial patches releasing EVs loaded with anti-fibrotic miRNAs effectively reverse myocardial remodeling. Local neuromodulation for cardiac autonomic regulation, achieved through focused ultrasound stimulation and local vagus nerve electrical stimulation, offers a novel non-pharmacological targeted strategy. This approach activates the cholinergic anti-inflammatory pathway locally, specifically inhibiting pro-fibrotic inflammatory communication between macrophages and fibroblasts in the injured myocardium. It avoids systemic autonomic side effects, representing a promising advancement in localized cardiac therapy.

### 8.6 Clinical translation challenges and future directions

Despite promising preclinical outcomes, translating communication-targeted anti-fibrotic therapies into clinical practice encounters significant obstacles (117). First, the heterogeneity of fibrosis—varying by etiology, stage, and cell network—diminishes the effectiveness of single-target treatments across diverse patient groups. Second, drug targeting and safety are concerns, as off-target effects of small-molecule inhibitors and RNA agents restrict their long-term use (118). Third, the absence of specific biomarkers hinders accurate staging, monitoring, and the guidance of precision therapies (119). To address these challenges, future research should prioritize several key areas. First, the creation of high-definition spatiotemporal cell communication maps using single-cell multi-omics and spatial transcriptomics is essential to identify core regulatory nodes for precise targeting (120). Second, developing cell-specific delivery systems, such as antibody-drug conjugates and engineered extracellular vesicles (EVs), can enhance targeting accuracy and minimize side effects. Third, identifying and validating fibrosis-specific biomarkers will facilitate early diagnosis and dynamic monitoring. Finally, conducting clinical trials that explore multi-target combination and sequential therapies is crucial for translating preclinical efficacy into clinical benefits (121). Decoding intercellular communication networks in cardiac fibrosis will not only propel the development of novel anti-fibrotic drugs but also establish a new theoretical framework for cardiovascular disease therapy.

### 8.7 Limitations of current included studies

Despite significant advancements in understanding intercellular communication networks in cardiac fibrosis, current studies face several critical limitations (122). Firstly, most findings are based on animal models (mice, rats, pigs), which fail to fully replicate the genetic diversity, pathological complexity, and comorbidities of human cardiac fibrosis. Secondly, many studies concentrate on individual signaling pathways, communication modes, or cell types, which do not capture the true multicellular and multifactorial nature of network regulation (123). Thirdly, while numerous studies highlight the pro-fibrotic or anti-fibrotic effects of specific molecules, they often overlook the spatiotemporal dynamics across different stages, such as acute injury, chronic remodeling, and irreversible fibrosis. Fourthly, the safety of emerging interventions remains inadequately assessed, with concerns like off-target effects in gene therapy, immunogenicity of extracellular vesicles, and tumorigenicity risks of stem cell therapy requiring long-term evaluation (124). Lastly, there is a significant gap in translating basic research into clinical practice, as evidenced by the scarcity of large-scale, multicenter, randomized controlled clinical trials to validate the efficacy and safety of proposed targets and strategies (125). These limitations underscore the need for future research to be more standardized, human-focused, and comprehensive to facilitate the clinical application of basic scientific advancements.

## 9 Conclusion

Myocardial fibrosis is a complex pathological process characterized by dynamic intercellular communication among various cell types, including cardiomyocytes, immune cells, endothelial cells, pericytes, and fibroblasts. This shift from single-cell to network-focused research highlights the importance of these interactions. Cardiomyocytes initiate stress signals, immune cells function as hubs for inflammation and repair, and vascular cells act as bridges between the vasculature and stroma. Their coordinated communication is crucial in determining the progression of fibrosis. Future research should leverage single-cell multi-omics and spatial transcriptomics to identify stage-specific core regulators. This approach will aid in developing multi-target combination and sequential therapies for clinical application. Understanding these networks will provide a theoretical foundation for precise anti-fibrotic therapies and interventions in cardiac remodeling.
